# Titanium Versus Autologous Bone-Based Cranioplasty: A Systematic Review and Meta-Analysis

**DOI:** 10.7759/cureus.39516

**Published:** 2023-05-26

**Authors:** Helen Capitelli-McMahon, Narvair Kahlar, Shafiq Rahman

**Affiliations:** 1 Plastic Surgery, Hull Royal Infirmary, Hull, GBR; 2 General Practice, Sandwell and West Birmingham Trust, Birmingham, GBR; 3 Plastic Surgery, Sheffield Teaching Hospitals NHS Foundation Trust, Northern General Hospital, Sheffield, GBR

**Keywords:** cranioplasty, bone, autologous, alloplastic, titanium

## Abstract

At present, there is no gold standard when looking at reconstructive evidence for cranioplasty with the use of autologous bone as well as other synthetic materials. Titanium has been considered recently as a good option due to its unique properties such as strength and biocompatibility. Numerous studies have previously compared titanium with autologous bone for cranioplasty yet no meta-analysis has been performed within the literature to provide guidelines for craniofacial surgeons.

A systematic review and meta-analysis were performed as per the Preferred Reporting Items for Systematic Reviews and Meta-Analyses (PRISMA) guidelines. A search of electronic information was conducted to identify all comparative studies of autologous bone vs. titanium implants in cranioplasty following a craniectomy. The primary outcomes were measured as re-operation rates and cosmesis, the secondary outcome measures included the incidence of complications, for example, bone resorption and infection.

Five studies were selected, enrolling 323 cases. A high reoperation rate (p > 0.007) was seen in autologous cranioplasty using bone due to the significantly high resorption rate reported in this group. Cosmetic outcomes demonstrated no significant difference between the two groups examined. Finally, costs and infection rates (p > 0.18) were found to be comparable.

Overall, titanium implants used in cranioplasty offer lower re-operation rates in comparison to autologous bone grafts whilst there was no major increase in adverse outcomes such as postoperative cost or rates.

## Introduction and background

Cranioplasty is a surgical procedure performed to restore a defect in the cranial vault after a previous decompressive craniectomy. This is usually following a traumatic brain injury, ischaemic or haemorrhagic disease, or after the removal of cranial tumours [1]. Cranioplasty is important in providing cerebral protection and improving cosmesis. It also serves to help control variations in cerebrospinal ﬂuid (CSF), blood ﬂow and the metabolic demands of the brain. Many different materials have been used to replace bony defects. These can be divided broadly into biological and synthetic. Autologous cranioplasty takes bone from either the cranial vault itself or elsewhere in the body. It has been a popular choice, being strong and biocompatible with low rejection rates [2]. However, the most common complications following the use of autologous bone grafts are infection and resorption, with the latter particularly leading to high rates of re-operation. This has been reported notably in the paediatric population, as well as in cases where the cranial defect is larger [3,4]. Synthetic materials have emerged to offer an alternative option combined with computer-based customisation and three-dimensional printing, which has reduced the operating time [2].

Titanium, in particular, has many advantages when compared to other synthetic materials. It offers superior cosmetic results, even in large defects [5]. It also has high biocompatibility and signiﬁcantly lower infection rates when compared to other popular synthetic alternatives such as polymethylmethacrylate (PMMA) [6,7].

Although the use of both autologous bone and titanium implants has been reported in randomised controlled trials (RCTs) and retrospective single-centre studies, there are currently no systematic reviews or meta-analyses that compare the use of titanium against autologous implants for cranioplasty following a craniectomy. This is, therefore, the ﬁrst study within the literature reporting on this topic. The authors aim to amalgamate the literature and enhance the existing evidence base on this topic so as to best guide the surgeon.

## Review

Methods

**Figure 1:**
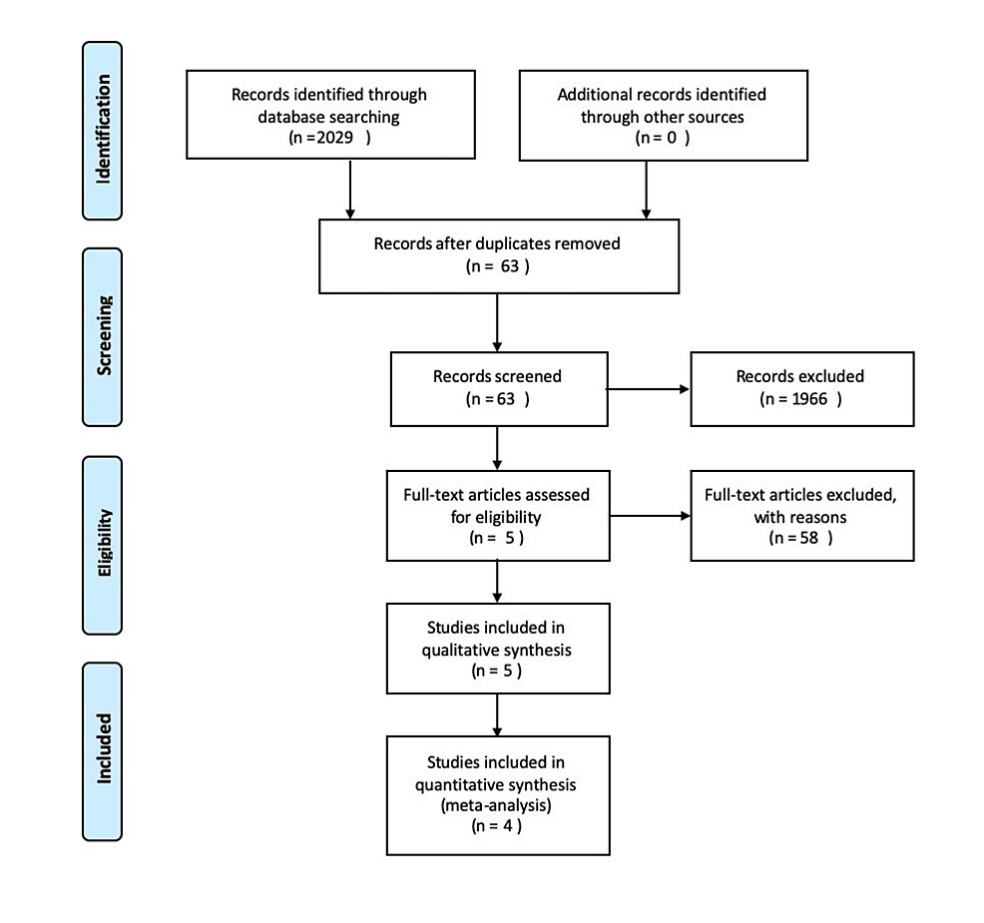
PRISMA flow diagram The Preferred Reporting Items for Systematic Reviews and Meta-Analyses (PRISMA) diagram details the search and selection processes applied during the overview.

This systematic review was performed according to an agreed predefined protocol in accordance with the Preferred Reporting Items for Systematic Reviews and Meta-Analyses (PRISMA) statement standards (Figure 1, Table 4).

Eligibility Criteria

The authors aimed to compare the use of autologous vs. titanium-based cranioplasty in all randomised control trials as well as observational studies. The use of titanium for cranioplasty was considered as the intervention of interest and autologous bone as a comparator. All patients were included regardless of age or co-morbidity status. Articles not reported in English, as well as those in which other synthetic materials apart from titanium were used, were excluded.

Outcome Measures

Re-operation rates and cosmesis were the primary outcome measures, and the incidence of complications such as bone resorption and infection were the secondary outcome measures.

Literature Search Strategy

Two authors (HCM and NK) independently searched the following electronic databases, including MEDLINE, EMBASE, CINAHL and the Cochrane Central Register of Controlled Trials (CENTRAL). The last search was run on 1 May 2020. The keywords and medical subject headings (MeSH) included “titanium cranioplasty”, “synthetic cranioplasty”, “autologous cranioplasty”, “autologous bone” and “craniectomy” These terms were combined with adjuncts of “and” as well as “or”. The authors also searched the bibliographic lists of relevant articles and reviewed them for further eligibility of articles.

Study Selection

Two authors (HCM and NK) independently assessed the abstract of articles identified from the searches. Full texts of the relevant articles were then obtained and those reports that met the eligibility criteria of our review were chosen. Any discrepancies in study selection were resolved by discussion between the authors. A third author (SR), who was independent, was consulted in the event of a disagreement.

Data Collection

We generated a digital data extraction spreadsheet in line with Cochrane's data collection form for intervention reviews. We pilot-tested the spreadsheet in randomly selected articles and adjusted it accordingly. Two authors (HCM and NK) independently obtained and finalised the data and resolved disagreements by discussion. If there was no agreement, a third author (SR) was consulted.

Methodological Quality and Risk of Bias Assessment

The two authors (HCM and NK) independently assessed the methodological quality and risk of bias of the articles matching the inclusion criteria. We used Cochrane's tool for assessing the risk of bias of randomised trials. Cochrane’s tool assesses domains, including selection bias, performance bias, detection bias, attrition bias, reporting bias and other sources of bias, and for each individual domain, classifies studies into low, unclear and high risk of bias. If no agreement could be reached, a third author (SR) acted as an adjudicator (Table 1, Table 2).

**Table 1:** Cochrane Collaboration’s tool Assessment of risk of bias of the randomised trials using Cochrane Collaboration’s tool

First Author	Bias	Authors’ Judgement	Support for Judgement
Honeybul 2017 [8]	Random sequence generation (selection bias)	Low Risk	Each participant was randomised according to a random sequence generated by a random number software
	Allocation concealment (selection bias)	Low Risk	Group allocation concealed.
	Blinding of participants and personnel (performance bias)	Unclear risk	No information given
	Blinding of outcome assessment (detection bias)	Unclear Risk	No information given
	Incomplete outcome data (attrition bias)	Low Risk	No missing data
	Selective reporting (reporting bias)	Low Risk	All outcome data reported
	Other bias	Low Risk	Similar baseline characteristics in both groups.
Honeybul 2018 [9]	Random sequence generation (selection bias)	Low Risk	Each participant was randomised according to a random sequence generated by a random number software
	Allocation concealment (selection bias)	Low Risk	Group allocation concealed.
	Blinding of participants and personnel (performance bias)	Unclear Risk	No information given
	Blinding of outcome assessment (detection bias)	Unclear Risk	No information given
	Incomplete outcome data (attrition bias)	Low Risk	No missing data
	Selective reporting (reporting bias)	Low Risk	All outcome data reported
	Other bias	Low Risk	Similar baseline characteristics in both groups

**Table 2:** Newcastle-Ottawa scale Newcastle-Ottawa scale to assess the quality of non-randomised studies * = reflects the quality of each section. The possible total points are 4 points for Selection, 2 points for Comparability, and 3 points for Outcomes

Study	Selection	Comparability	Exposure
Liang [10]	***	**	***
Brougton [11]	***	**	***
Piitulainen [12]	**	*	***

Data Synthesis and Statistical Analyses

We planned to perform a meta-analysis of the outcomes reported by at least three studies. For dichotomous outcome variables, we planned to calculate the odds ratio (OR). The OR is the odds of an event in the biological group compared to the non-biological group. For continuous parameters, we planned to calculate the mean difference (MD) between the two groups. The authors aimed to use Review Manager 5.3 software (available at revman.cochrane.org for data synthesis with a random effects model; The Cochrane Collaboration, London, UK). All results were aimed to be reported in a forest plot with 95% confidence intervals (CIs). The heterogeneity among the studies was assessed with the Cochran Q test (χ2). We also planned to quantify inconsistency by calculating I2 and interpreting it using the following guide: 0% to 25% may represent low heterogeneity; 25% to 75% may represent moderate heterogeneity; 75% to 100% may represent considerable heterogeneity.

Results

Literature Search Results

Our search strategy retrieved 63 studies, and after a thorough screening of retrieved articles, the authors identified five studies in total which met the eligibility criteria (Figure 1). Fifty-eight full-text articles were excluded, as these studies either did not compare the two groups, did not measure the same outcome or did not meet the criteria. The baseline characteristics of the included studies are summarised in Table 3.

**Table 3:** Baseline characteristics of the included studies

Study	Year	Country	Journal	Study Design	No. of participants in the control + intervention group	Cranioplasty operations compared
Honeybul [8]	2017	Australia	Journal of Neurosurgery	Randomised Controlled Trial	64	Autograft, titanium
Honeybul [9]	2018	Australia	Acta Neurochirurgica	Randomised Controlled Trial (follow-up)	62	Autograft, titanium
Liang [10]	2015	New Zealand	British Journal of Neurosurgery	Single Centre Retrospective Study	88	Autograft, titanium, acrylic, polyetheretherketone (PEEK)
Broughton [11]	2014	UK	British Journal of Neurosurgery	Single Centre Retrospective Study	87	Autograft, titanium, acrylic
Piitulainen [12]	2015	Finland	World Neurosurgery	Single Centre Retrospective Study	100	Autograft, bioactive fibre-reinforced composite, hydroxyapatite, other synthetic materials

Description of Studies

Honeybul et al. (2017) and Honeybul et al. (2018) [8,9]: Honeybul et al. conducted a prospective randomised controlled trial, which included 64 patients who underwent decompressive craniectomies and had their own bone available for subsequent cranioplasty procedures. Patients were randomised to receive either their own bone or a primary titanium cranioplasty. Outcome measures included implant failure rate, adverse events, cosmetic and functional outcomes and total costs. Honeybul followed up patients in the initial study for a minimum of 24 months, publishing results in a second paper [9]. Sixty-two patients were included in this retrospective follow-up cohort.

Liang et al. (2015) [10]: This single-centre retrospective observational study reports cranioplasty outcomes and evaluates the factors involved in their management. Eighty-eight (88) patients were included in this study, 53 had autologous cranioplasty, 17 had titanium cranioplasty, with the other patients receiving acrylic or polyetheretherketone (PEEK). There was a standard follow-up period of three months. Indications for surgery and patient co-morbidities and complications were recorded.

Broughton et al. (2014) [11]: This retrospective observational study evaluated indications, techniques and outcomes for 87 patients undergoing cranioplasty at a single centre. Twenty-six per cent (26%) of patients underwent autologous cranioplasty, with titanium being the most common synthetic implant (53% of patients).

Piitulainen et al. (2015) [12]: Piitulainen et al. conducted a single-centre retrospective review of 84 patients, with 20 patients undergoing autologous cranioplasty and others undergoing cranioplasty with fibre-reinforced composite, HA bone cement paste or other synthetic implants, including nine patients receiving a titanium plate.

Primary Outcomes

Re-operation rates: In Honeybul et al., seven of 31 patients (22%) in the autologous cranioplasty group underwent re-operation due to significant bone resorption causing loss of cerebral protection [8]. Five patients initially underwent secondary cranioplasty with a titanium plate. On longer-term follow-up, Honeybul et al. reported that two patients with initial bone resorption underwent re-operation after 12 months for functional and cosmetic reasons [9]. Another patient initially noted to have moderate bone resorption re-presented one year later with progressive flap resorption and required augmentation for functional and cosmetic reasons. A total of eight patients underwent re-operation in the autologous bone group but none in the titanium group.

Piitulainen et al. reported that eight of 20 patients (40%) in the autologous cranioplasty group underwent further surgery [12]. Three patients had significant bone resorption (15%) and five presented with surgical site infections (25%) requiring re-operation. None of the patients undergoing cranioplasty with titanium plate underwent further procedures in the three-month follow-up.

**Figure 2:**
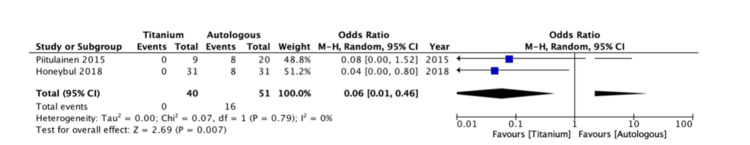
Re-operation rate [8,9,12]

In Figure 2, the re-operation rate was reported in three studies enrolling 91 patients. Two studies were included in the analysis as the Honeybul [9] follow-up study included the same patient group as the initial Honeybul group [8]. There was a statistically significant difference showing the titanium group to have a lower re-operation rate compared to the autologous group (CI = 0.01 to 0.46, P < 0.007). A low level of heterogeneity was found among the studies (I2 = 0%, P = 0.79).

Cosmesis: Honeybul et al. report the cosmetic result from both a patient and clinician perspective [8]. Outcomes were similar for both, with 23 patients in the autologous group and 31 patients in the titanium group achieving satisfactory, partial or complete success when assessing cosmetic outcomes. Broughton et al. recorded six patients with poor cosmesis [11]. Of these, two were autologous bone and two were titanium plates.

Secondary Outcomes

Bone resorption: Bone resorption in the autologous group was a significant factor in patients requiring re-operation (see 3.3.1). Overall, three studies noted bone resorption rates. Piitulainen et al. [12] described three cases, and Honeybul et al. [8,9] noted a total of eight cases on long-term follow-up.

**Figure 3:**
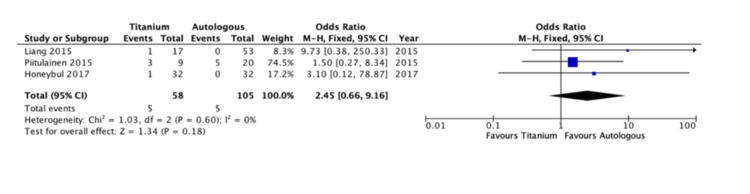
Infection rates [8,10,13]

Infection rates: The infection rate following the autologous and titanium cranioplasty operations was reported in three studies enrolling 163 patients (Figure 3) [8,10,13]. There was no significant difference between the two groups (CI = 0.66 - 9.16, P <0.18). A low level of heterogeneity was reported (I2 = 0%, P = 0.60).

Other Complications

One study included data on long-term seizure activity, with five patients developing seizures following autologous cranioplasty compared to three patients in the titanium group. This was not statistically significant. One study included the development of postoperative haematoma, however, did not include which type of cranioplasty this followed.

Discussion

Autologous bone grafting is cheap, strong and biocompatible. However, bone resorption is a significant caveat. Honeybul et al. reported that 22% of patients underwent complete resorption such that their operation was reported as an overall failure [8]. Similarly, Piitulainen et al. found bone resorption responsible for 15% of graft removal [12]. This was seen particularly in younger patients (p = 0.013). In the follow-up study by Honeybul et al., seven patients underwent further surgery due to bone resorption [9], and this also negatively impacted cosmetic results. Titanium implants did not report such complications.

Titanium is a non-ferrous inert metal, which when compared to other synthetic cranioplasty options has a low long-term failure rate [13]. This correlates with its low post-operative infection rates, which has led to titanium being a popular choice of material for post-craniotomy infections [14,15], as well as its use for primary cranioplasty following craniectomy.

Titanium is strong, with high biocompatibility and excellent handling characteristics. This means it protects the brain from potential trauma, and with the input of computer-based customisation, has meant that titanium has superior cosmetic outcomes when compared to other synthetic options [5]. Additionally, titanium is relatively radiolucent, which allows for clear images on CT and MRI post-operatively [15].

Other synthetic materials have reported complications, with Piitulainen et al. identifying PMMA to be associated with higher rates of graft infection and displacements, leading to implant removal [12]. Although HA showed promising results due to low complication rates, its low mechanical strength makes it unsuitable for larger defects, unlike titanium plating.

The use of titanium plating for cranioplasty following craniectomy was superior to autologous cranioplasty when considering the rate of re-operation. There was a significant (P < 0.007) difference between those requiring further operations in the autologous group as compared to the titanium group (Figure 2). This is related to bone resorption in the autograft group as the most common reason for re-operation. Honeybul et al. reported a 22% re-operation rate, all cases due to bone resorption causing a loss of cerebral protection [9].

Conversely, there were no significant differences (P <0.18) in the analysis of post-operative infection rates between the two groups (Figure 3). In terms of the between-study heterogeneity, it was low for both outcomes (I2 = 0%).

There was inconsistency in reporting cosmetic outcomes among the studies. Honeybul et al. and Broughton et al. reported similar cosmetic outcomes, with no significant difference between the two groups [8,11]. Liang et al. reported eight cases of poor cosmetic results but did not detail in which groups or if further treatment was required [10]. Honeybul et al. have broken down the costs incurred for both autologous bone and titanium implants [8]. Titanium plates had a greater initial investment, averaging $3,500 per patient compared to $547 per patient for autologous bone grafts. However, the total cumulative amount spent between the two groups was not significantly different (p = 0.327) due to extra costs incurred through complications following bone resorption. Long-term costs for titanium implants may be lowered with the advances in computer-assisted design and manufacturing (CAD/CAM), allowing prefabricated plates to be custom-made [5].

Limitations

This meta-analysis has limitations. Only ﬁve papers were included in this analysis, with only one randomised controlled trial. The majority of studies included were observational in design with low scores for comparability on the Newcastle-Ottawa scale. The overall number of titanium implants considered is relatively small. Honeybul et al. followed up with patients for 24 months [9]; however, other studies included had shorter follow-up periods. Longer follow-up periods, as well as a more detailed study of complications seen in younger populations compared to older, more co-morbid patients, will help identify the optimum material for each group. The authors suggest more high-quality randomised control trials, including an assessment of paediatric patients. In addition, this meta-analysis excluded articles that were not published in English. This may skew our outcome data, as many lower to middle-income countries do not have expensive protocol-driven standards of care for the treatment of a traumatic brain injury (TBI) as higher-resource settings. These centres may, therefore, still favour autologous cranioplasty options where titanium and other synthetic choices are not readily available and studies from these areas may have been missing from our study [16].

## Conclusions

Although the evidence is limited, with only one randomised-controlled trial and three observational studies comparing autologous to titanium implants, the results of this meta-analysis suggest that the re-operation rate is lower where titanium implants are used, due to rates of bone resorption causing loss of cerebral protection in autologous grafting. Rates of other post-operative complications, particularly infection, were not statistically different between the two treatment groups. The authors suggest more randomised clinical trials to further the current evidence base and more accurately record cosmetic complications between the two techniques. Based on the current evidence published, titanium appears to be a more suitable option, however, it is important to note that all reported studies were from developed countries and the authors would encourage the publication of data from lower economic countries to assess how their practices and outcomes vary before establishing definitive guidelines.

## Appendices

**Table 4:** PRISMA checklist Preferred Reporting Items for Systematic Reviews and Meta-Analyses

Section and Topic	Item #	Checklist item	Location where item is reported
TITLE			
Title	1	Identify the report as a systematic review.	1
ABSTRACT			
Abstract	2	See the PRISMA 2020 for Abstracts checklist.	1
INTRODUCTION			
Rationale	3	Describe the rationale for the review in the context of existing knowledge.	1
Objectives	4	Provide an explicit statement of the objective(s) or question(s) the review addresses.	1
METHODS			
Eligibility criteria	5	Specify the inclusion and exclusion criteria for the review and how studies were grouped for the syntheses.	2
Information sources	6	Specify all databases, registers, websites, organisations, reference lists and other sources searched or consulted to identify studies. Specify the date when each source was last searched or consulted.	2
Search strategy	7	Present the full search strategies for all databases, registers and websites, including any filters and limits used.	2
Selection process	8	Specify the methods used to decide whether a study met the inclusion criteria of the review, including how many reviewers screened each record and each report retrieved, whether they worked independently, and if applicable, details of automation tools used in the process.	2
Data collection process	9	Specify the methods used to collect data from reports, including how many reviewers collected data from each report, whether they worked independently, any processes for obtaining or confirming data from study investigators, and if applicable, details of automation tools used in the process.	3
Data items	10a	List and define all outcomes for which data were sought. Specify whether all results that were compatible with each outcome domain in each study were sought (e.g. for all measures, time points, analyses), and if not, the methods used to decide which results to collect.	4-5
	10b	List and define all other variables for which data were sought (e.g. participant and intervention characteristics, funding sources). Describe any assumptions made about any missing or unclear information.	4-5
Study risk of bias assessment	11	Specify the methods used to assess risk of bias in the included studies, including details of the tool(s) used, how many reviewers assessed each study and whether they worked independently, and if applicable, details of automation tools used in the process.	3-4
Effect measures	12	Specify for each outcome the effect measure(s) (e.g. risk ratio, mean difference) used in the synthesis or presentation of results.	5-6
Synthesis methods	13a	Describe the processes used to decide which studies were eligible for each synthesis (e.g. tabulating the study intervention characteristics and comparing against the planned groups for each synthesis (item #5)).	4
	13b	Describe any methods required to prepare the data for presentation or synthesis, such as handling of missing summary statistics, or data conversions.	4-5
	13c	Describe any methods used to tabulate or visually display results of individual studies and syntheses.	5
	13d	Describe any methods used to synthesize results and provide a rationale for the choice(s). If meta-analysis was performed, describe the model(s), method(s) to identify the presence and extent of statistical heterogeneity, and software package(s) used.	5
	13e	Describe any methods used to explore possible causes of heterogeneity among study results (e.g. subgroup analysis, meta-regression).	5-6
	13f	Describe any sensitivity analyses conducted to assess robustness of the synthesized results.	5-6
Reporting bias assessment	14	Describe any methods used to assess risk of bias due to missing results in a synthesis (arising from reporting biases).	3
Certainty assessment	15	Describe any methods used to assess certainty (or confidence) in the body of evidence for an outcome.	3
RESULTS			
Study selection	16a	Describe the results of the search and selection process, from the number of records identified in the search to the number of studies included in the review, ideally using a flow diagram.	4-5
	16b	Cite studies that might appear to meet the inclusion criteria, but which were excluded, and explain why they were excluded.	4
Study characteristics	17	Cite each included study and present its characteristics.	4-5
Risk of bias in studies	18	Present assessments of risk of bias for each included study.	3-4
Results of individual studies	19	For all outcomes, present, for each study: (a) summary statistics for each group (where appropriate) and (b) an effect estimate and its precision (e.g. confidence/credible interval), ideally using structured tables or plots.	5-6
Results of syntheses	20a	For each synthesis, briefly summarise the characteristics and risk of bias among contributing studies.	3
	20b	Present results of all statistical syntheses conducted. If meta-analysis was done, present for each the summary estimate and its precision (e.g. confidence/credible interval) and measures of statistical heterogeneity. If comparing groups, describe the direction of the effect.	5-6
	20c	Present results of all investigations of possible causes of heterogeneity among study results.	5-6
	20d	Present results of all sensitivity analyses conducted to assess the robustness of the synthesized results.	5-6
Reporting biases	21	Present assessments of risk of bias due to missing results (arising from reporting biases) for each synthesis assessed.	3
Certainty of evidence	22	Present assessments of certainty (or confidence) in the body of evidence for each outcome assessed.	3
DISCUSSION			
Discussion	23a	Provide a general interpretation of the results in the context of other evidence.	6-7
	23b	Discuss any limitations of the evidence included in the review.	6-7
	23c	Discuss any limitations of the review processes used.	6-7
	23d	Discuss implications of the results for practice, policy, and future research.	6-7
OTHER INFORMATION			
Registration and protocol	24a	Provide registration information for the review, including register name and registration number, or state that the review was not registered.	7
	24b	Indicate where the review protocol can be accessed, or state that a protocol was not prepared.	7
	24c	Describe and explain any amendments to information provided at registration or in the protocol.	7
Support	25	Describe sources of financial or non-financial support for the review, and the role of the funders or sponsors in the review.	7
Competing interests	26	Declare any competing interests of review authors.	7
Availability of data, code and other materials	27	Report which of the following are publicly available and where they can be found: template data collection forms; data extracted from included studies; data used for all analyses; analytic code; any other materials used in the review.	7
